# First Complete Genome Sequence of a Watermelon Mosaic Virus Isolated from Watermelon in the United States

**DOI:** 10.1128/genomeA.00299-16

**Published:** 2016-04-21

**Authors:** Naveen Rajbanshi, Akhtar Ali

**Affiliations:** Department of Biological Science, The University of Tulsa, Tulsa, Oklahoma, USA

## Abstract

Watermelon mosaic virus was first reported in 1965 from the Rio Grande Valley, TX. We report here the first complete genome sequence of a watermelon mosaic virus isolate from watermelon collected from the Rio Grande Valley of Texas.

## GENOME ANNOUNCEMENT

Watermelon mosaic virus (WMV) (genus *Potyvirus*, family *Potyviridae*) occurs worldwide and is economically important due to its effects on losses in cucurbit crops (1). Cucurbits are economically important cash crops in the United States and are mostly grown in the southern states, but Texas and Florida are the two major watermelon-producing states (2).

WMV was first reported in the United States in the Rio Grande Valley of Texas in 1965 (3, 4). Since then, it has been recognized as a major virus infecting cucurbits in several states, such as Illinois (5), New Jersey (6), California (7), and Oklahoma (8, 9). In our previous study, WMV was the most common virus detected in 9 out of 10 southern states, with the highest average incidence of 30.6% among 17 viruses tested (10).

Despite the importance of WMV, no complete genome of WMV from cucurbit crops has ever been sequenced in the United States, although partial coat protein sequences are available. Here, we present the first complete genome sequence of a WMV isolate from watermelon collected in a commercial watermelon field in South Texas.

During our previous surveys of cucurbits (10), a number of WMV-positive samples were collected in the Rio Grande Valley, TX. One of the WMV-positive watermelon samples, known as TX29, showed severe symptoms and was selected for this study. The TX29 isolate was propagated by mechanically inoculating squash seedlings using extracted sap from infected watermelon leaves collected in the field.

Total RNA was extracted from the leaf of a squash seedling infected with the WMV TX29 isolate, according to the Tri-Reagent procedure (8). Nine pairs of overlapping primers were designed from the complete genome sequences of WMV isolates available from GenBank (http://www.ncbi.nlm.nih.gov). Nine genome fragments were amplified by reverse transcription-PCR (RT-PCR), with the respective primer pairs, using total RNA as the template, as described previously (8). PCR products were confirmed on 1% agarose gels, cloned into pGEM-T Easy vector, and three to five recombinant clones were sequenced in both directions using Applied Biosystems 3130 at the Department of Biological Science, The University of Tulsa, OK.

Sequence alignment was done using EditSeq and MEGAlign within the DNAStar suite of programs (Madison, WI, USA). Consensus nucleotide sequences were obtained using the Clustal X program (11). The complete genome was generated by joining the overlapping sequences among the adjacent fragments. Phylogenetic analysis among the various sequences of WMV isolates was inferred by the maximum-likelihood method using the general time reversible (GTR) + G module in MEGA5 with 1,000 bootstrap replicates (12).

The complete genome sequence of the TX29 isolate is 10,049 nucleotides long, excluding the 3′ terminal poly(A) tail. Phylogenetic analysis showed that the WMV TX29 isolate shared 89 to 97% nucleotide and 88 to 98% amino acid sequence identity with the published WMV isolates. Maximum nucleotide (97%) and amino acid (98%) sequence identity was observed with a French isolate (FMF03-141, accession no. EU660583) (13).

### Nucleotide sequence accession number.

The complete genome sequence of the WMV TX29 isolate was deposited in GenBank under the accession no. KU246036.
